# Detecting Soil Microarthropods with a Camera-Supported Trap

**DOI:** 10.3390/insects11040244

**Published:** 2020-04-14

**Authors:** Norbert Flórián, Laura Gránicz, Veronika Gergócs, Franciska Tóth, Miklós Dombos

**Affiliations:** Institute for Soil Sciences and Agricultural Chemistry, Centre for Agricultural Research, Herman Ottó út 15, H-1022 Budapest, Hungary; laura.granicz@gmail.com (L.G.); gergocs.veronika@agrar.mta.hu (V.G.); franciska.toth19@gmail.com (F.T.); dombos.miklos@agrar.mta.hu (M.D.)

**Keywords:** mesofauna, automatic detection, wireless sensor, infrared sensor, sampling method, activity, monitoring system, Collembola, Acari

## Abstract

There is an increasing need to monitor activity and population growth of arthropods; however, this is a time-consuming and financially demanding process. Using sensors to detect arthropods in the field can help to follow their dynamics in time. Improving our earlier device, we developed a new camera-supported probe to detect soil microarthropods. An opto-electronic sensor ring detects the caught microarthropod individuals what activates a camera. The camera takes pictures of a specimen when it arrives in the camera chamber. A vacuum device was built into the probe which pumps up the specimen from the probe to a sample container. Here, we describe the construction and operation of the probe. We investigated the precision of the process in a laboratory experiment using living microarthropods and evaluated the accuracy of the probes in a semi-natural investigation when environmental noise was present. Under semi-natural conditions, the percentages of success, i.e., the photographed specimens compared to the caught ones, were between 60% and 70% at the investigated taxa. The automatic camera shooting helped in distinguishing insects from irrelevant detections while collecting the trapped insects allowed species-level determination. This information together serves as a basis for the automatic visual recognition of microarthropod species.

## 1. Introduction

Soil mesofauna, mainly Collembola and Acari, play a vital role in regulating and enhancing nutrient cycling and decomposition and are represented in a high number and diversity in most natural and semi-natural habitats. These animals live hidden in the soil; therefore, our knowledge about their ecological features and roles is still limited.

In soil ecological studies, the population size of soil mesofauna is usually characterized by two measures. First, the density counts the individuals by taking a certain amount of soil and extracting them from it [1]. Second, the activity-density sampling mainly monitors mobile and surface-living (epigeic) species [2]. This measure indicates the number of specimens trapped in a given period, and it correlates with the abundance and activity of the focal population [3]. Pitfall trapping is the most widely used method for trapping surface-living species; however, several drawbacks of the method have been recognized [4,5]. For pitfall trapping, a simple plastic cup is buried into the soil surface. Insects moving on the soil surface fall into the cup and are killed by a preservation liquid. To enhance sampling efficiency, improved approaches such as cul-de-sac traps, basket traps, and others [1,6] exist; however, these methods are still not widespread.

In the last decades, several claims have been formulated about trap designs to achieve higher representability, efficiency, and lower disturbance. The new probe was designed to meet the following criteria: (1) Trap devices should operate autonomously for extended periods of time; (2) reduced costs and human effort for studying temporal patterns of microarthropods; and (3) bias should be kept as low as possible. While today a variety of designs are available [7], each has some drawbacks. Thus, it is being increasingly recognized that existing methods should be combined, supplemented, or run side-by-side to obtain better and less biased results [8].

To better understand the role of arthropods in a given ecosystem, the timing of their activity needs to be specified in addition to their total abundance and diversity. The activity of microarthropods is influenced by environmental (e.g., humidity, frost) and biological factors (e.g., finding suitable food, searching for mating partners, avoiding predators) [9,10]. Moreover, their activity may influence soil processes [11]. To measure the activity and to provide more accurate real-time data, vast effort has been made in the field of arthropod detection. Nowadays, these studies are advancing to detect flying and surface-living arthropods, mainly macroarthropods, using camera traps and opto-electronic or acoustic sensors [12,13,14,15,16]. However, such detection methods are not applicable for soil and soil surface dwelling microarthropods, because of the small size and cryptic life of the latter.

Animal activity is usually followed by different types of camera traps. Some of them are popular in vertebrate ecology. Security IT cameras have been applied to turn on cameras in traps that have been developed for warm-blooded organisms using a passive infrared sensor. Another way would be to use motion detection cameras, but their power consumption is so high that they need a wired power supply [17]. Time-sorting pitfall traps [16,18], videos [8], and time-lapse videos for arthropods of larger sizes [19] are the most promising methods, but these are still not widely used. In addition, these methods provide data in different resolutions of time (e.g., daily or every 15 minutes), but not continuously, and they are often energy demanding and only suitable to run for a short time or indoors.

Previously, the so-called EDAPHOLOG probe was developed to reduce energy consumption and human effort and to gain more information about the activity of soil-dwelling microarthropods [20]. In this probe, an infrared (IR) sensor was optimized for detecting soil-living microarthropods [21]. The previous version of EDAPHOLOG had several small drawbacks that need to be solved and corrected. Although the probes had good potential in capturing the members of soil mesofauna and in detecting the activity of soil-dwelling and surface-living microarthropods [20], soil particles falling into the probe caused a notable bias in detection. A significant difference was found between the captured and detected numbers of soil microarthropods. Therefore, a data filter algorithm was used to select false detections. In this way, however, data of smaller microarthropods were lost. In addition, because of its physical constraints, the probes had to be dug down deep into the soil; therefore, the whole probe had to be taken out of the soil while checking the sample containers. These actions caused considerable disturbances in the microhabitat around the probes. Moreover, soil particles that fell in easily obstructed the probes, so they had to be checked quite often (monthly).

Based on EDAPHOLOG, we built an advanced, camera-supported trapping probe that automatically takes pictures of each detected specimen, aiming to distinguish between captured microarthropods and soil particles falling into the trap. Besides that, automatic camera shooting has the potential advantage of identifying morphotypes and higher taxa of microarthropods. We modified the earlier probe in size and shape to optimize the photo-shooting system. When the microarthropods fell into the camera chamber of the trap, an infrared sensor ring activated the built-in camera. The infrared sensor ring used in the current probe has already been described and tested by Balla, et al. [22]. The captured animals were actively moved out from the camera chamber towards a sample container after photo shooting. In this paper, we describe the physical construction of the camera-supported probe and show the precision and accuracy of the whole sampling process under laboratory conditions.

## 2. Materials and Methods

### 2.1. Description of the Probe

**Figure 1:**
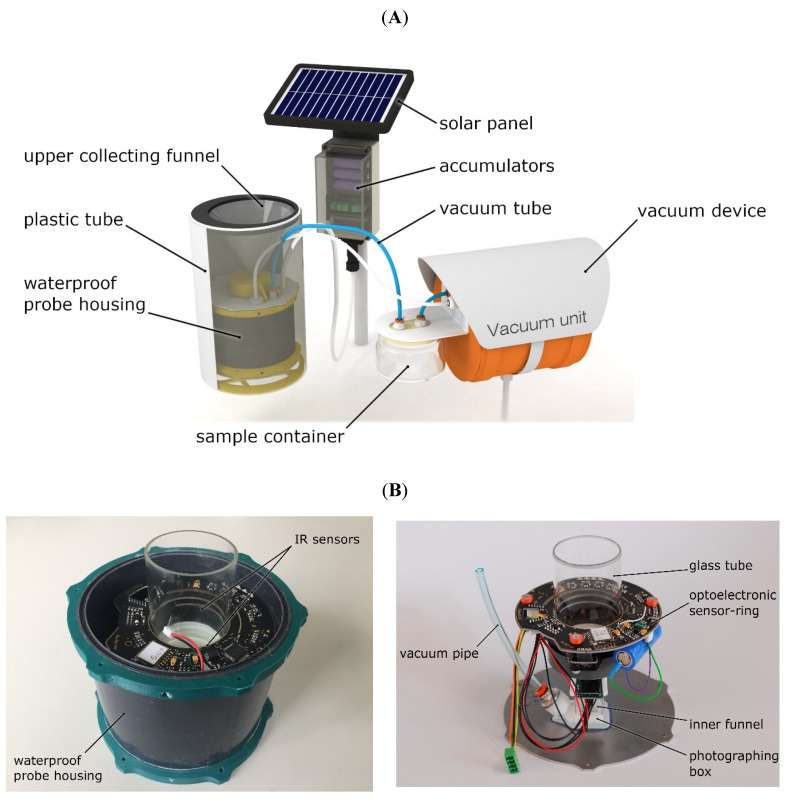
Construction of the new probe. (**A**) Construction of the camera-supported probe and the joint system (pneumatic unit and data logger with electric supply). (**B**) Photos of the probe, inside the protecting tube (left side) and components taken out from the protecting tube (right side).

The new probe consisted of a collecting funnel joined to the top of a waterproof probe housing (Figure 1). These parts can be placed in a plastic tube (diameter = 15 cm), which, in the future, will be deployed in the field. The plastic housing allows the probe to be removed without disturbing the soil structure. A vacuum device was built into the system, which can pump up the specimens from the probe to a sample container. The sample container can be placed aboveground and connected by a vacuum tube to the probe. Thus, it will not be necessary to remove the probe from the soil when collecting the biological sample. A logger was attached to the probe, which transmits data to the server and supplied electric power with the help of accumulators and a solar panel (Figure 1). 

The funnel was covered with a net (mesh size: 2 × 2 mm) to keep larger fragments and animals out. Thus, the collecting funnel can be placed flush with the soil surface or directly below the O soil horizon. Detection was initialized when a specimen fell into the trap. It first fell through the collecting funnel, then through a glass tube and entered the photographing box (see Figure 1B). The detection of a fall-in was generated by an opto-electronic sensor ring that was placed around the glass tube. When the infrared optical sensor detected a fall-in, it activated a camera module. The camera took pictures of the captured specimen when it arrived in the photographing box. The opto-electronic sensor recorded only those events when the path between the receiver and the emitter was interrupted. It merely detected the arthropods falling through the sensor field. We used the infrared sensor ring 2 (IRSR-2) [22], which has a narrow sensor field, thereby detecting arthropods of small size. Closure of the probe was ensured by connecting the stainless-steel sheet and the plastic tube with waterproof rubber rings, both at the upper and lower part and at the glass tube in the detecting band. The probe could be easily disassembled to service and clean (Figure 1B).

**Figure 2:**
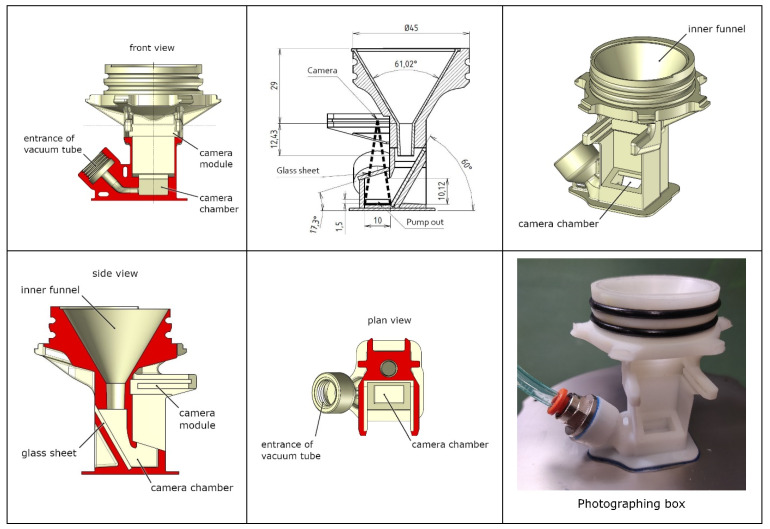
Blueprints of the photographing box (front and side views). Microarthropods fall into the inner funnel and enter the camera chamber. After the photo shooting, the specimen is pumped out from the camera chamber through a plastic pipe.

The photographing box was made of plastic via 3-dimensional printing. Under the sensor ring, the glass tube ended in a 60-degree funnel, which had an 8 mm outlet towards the camera chamber (Figure 2). The camera chamber was covered with a glass plate, above which the camera was mounted. The photography area is 13.3 × 10 mm, and the camera chamber has a 10 mm height and a rectangular opening of 13.3 × 5.7 mm (Figure 2).

When the IR optical sensor detected a fall-in, it activated the camera module. The µCAM-II-type camera module produced by 4D SYSTEMS took a picture of the specimen when it arrived at the camera chamber. The module used a CMOS variable gain amplifier color sensor along with a JPEG compression chip, and this provided a low-cost and low-power camera system. The module had an on-board serial interface that was suitable for a direct connection to any host microcontroller UART or a PC system COM port. The turn-on process was set to 3 seconds, leaving enough time for the specimen to fall into the camera chamber. Moreover, when sensors were initiated, motion detection started. When motion was detected or the time frame (3 seconds) expired, the camera took a picture and turned off automatically. For minimizing power consumption, the camera automatically set itself to standby after 15 seconds of inactivity. After detection and initialization, a photo was taken with 640 × 480 pixels, resulting in a 0.021 mm/pixel resolution.

As microarthropods may stay a long time in the camera chamber, they had to be moved actively out of it to make space for other specimens. After photography, the specimens were pumped up into the sample container through a plastic pipe (inner diameter: 4 mm). The vacuum unit sucks out the animals with −0.7–−0.5 bar.

The low power consumption IR sensor was permanently switched on, while the higher power consumption camera turned on only when it was recording and sending the upset file to the central processing unit. The central processing unit stored the pictures temporarily in EEPROM memory. The stored data were conveyed to the central data server by GSM/GPRS communication. Further information on the electronic construction of the camera is discussed in Supplement 1.

The power consumption of the device was low. It was supported by an electric panel, which supplied electricity by six pieces with Li-ion battery packs and a solar panel. It served as temporary data storage as well.

The system was designed for online monitoring, including its own data forwarding system, a central database, and a Web interface. A logger was connected to each probe and transmitted the sensor data to a central database via the internet each day (according to settings). Detected data, including pictures and time data, can be downloaded from or managed directly on the ZooLog Online web interface: https://helion.hu/zoolog/. The price of the prototype currently is around 1250 Euro. In the future large scale manufacturing is likely reduce the price

### 2.2. Tests

**Figure 3:**
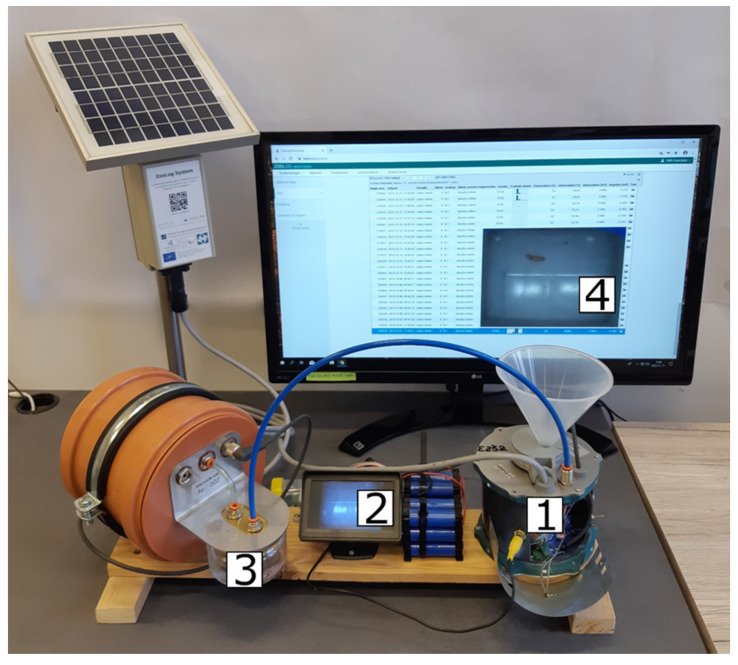
Testing the sensor processes on a desktop model of the probe. (**1**) Detection of the fall-in of the microarthropod by the infrared sensor ring. (**2**) Checking if the specimen arrived at the camera chamber or not. (**3**) Checking if the vacuum unit could pump out the specimen from the camera chamber to the sample container. (**4**) Checking if the microarthropod can be seen in the photo and can be identified.

We tested the efficiency of the new camera-supported trap design. First, in examining the entire process, we built a desktop model (Figure 3) in which we attached a monitor to follow what happened with the specimens in the photo chamber. We dropped living microarthropods (0.47–2.47 mm) into the probes and measured the efficiency of each part of the process: Detection, access to the camera chamber, photo shooting, and pumping out with the vacuum device. Second, by testing the probes under semi-natural conditions, we set the device under a Berlese soil extractor, extracted live microarthropods from soil samples, and analysed the catches. Then, we compared the number of individuals from the biological samples to the data received on the ZooLog Online web interface.

#### 2.2.1. Process Test

We put living animals into the probes and tested the following: 1. Detection accuracy, 2. arriving into the camera chamber, 3. photo-shooting, and 4. vacuum efficiency (Figure 3).

We used three different microarthropod species kept in the laboratory: two collembolans, *Folsomia candida* Willem (Collembola, Isotomidae) and *Heteromurus nitidus* Templeton (Collembola, Entomobryidae), and one mite species, *Hypoaspis aculeifer* Canestrini (Mesostigmata, Laelapidae). Besides that, we used wild oribatid mites extracted from mixed forest leaf litter. After extraction, the animals were investigated within three days. Immediately prior to the tests, the body length of the microarthropods was measured by a CollScope device [23]. Specimens were dropped into the collecting funnel; then, each element of the whole process was recorded.

##### Detection Accuracy

In previous work, we tested the detection accuracy of separated sensor rings [22]. The parameter “detection threshold” set the sensitivity of the sensor, by which the sensing process was fitted to the body size of the arthropod (smaller-sized microarthropods need a lower “detection threshold”). For our recent study, we used a detection threshold of the setting parameter: 100 because it was proven to be more useful when the environmental noise became stronger [22], particularly under field conditions. Body lengths of specimens (50 individuals) of each test species were measured (ranging from 0.47 to 2.47 mm), and then the animals were individually dropped into the trap to record their detectability (yes or no). Each specimen was used only once.

##### Microarthropods Getting into the Camera Chamber

After detection, microarthropods fell into the camera chamber. We recorded whether the specimen was visible on the screen or not from the time of detection until the photo shooting. In the desktop model, a small monitor was attached to the camera, on which we could see what was happening in the camera chamber. Potential failure could be that specimens were stuck somewhere in the outer or inner funnel, or even before initialization of the camera they immediately fell through the camera chamber.

##### Efficiency of Photo Shooting

The camera was activated with some delay after detection. When the photo-shooting system was on, it started to operate and made one picture within 2-3 seconds. This time interval was investigated to determine whether it was enough for the captured specimen to move forward into the plastic tube or upward to those sides of the photo chamber where the camera was blind. We recorded if the specimen was visible in the picture or not.

##### Efficiency of Vacuum

The vacuum device pumped out the specimens into the sample container, which can be placed aboveground. For testing the efficiency of the vacuum device in the desktop model, the camera chamber was watched in real time, and whether the pumping-out event occurred after a given detection or not was checked. Finally, we recorded whether the specimens got into the container or not after a pumping-up event.

#### 2.2.2. Testing the Trap Systems under Semi-Natural Conditions

An experiment was also run to study the efficacy of the trap system under semi-natural conditions with specimens captured in the field. To achieve this, soil and leaf litter samples (about 3 litres) were collected from different habitats of an oak and a black locust woodland (near Budapest and Őrbottyán, Hungary). These samples were put into a Berlese funnel, and soil and litter living mesofauna were extracted from it. Within two days, the extracted arthropods were transferred to another previously defaunated soil (Calcic Cernic Chernozem, WRB) layer, samples were placed again in the Berlese extractor and used for the semi-natural experiment. Specimens from our laboratory cultures mentioned above (three species, see in Table 1) were also added to the soil layer. The probes were installed under the Berlese funnels, and these soil samples were extracted with a known number of soil-living fauna. Sometimes soil particles fell with the animals as well, similar to natural conditions. Thirteen probes were used in different time periods. Each test lasted four days. The initial number of arthropods was compared to the number of specimens gained back in the sample container. The number of photo-captured arthropods was also compared with those that were obtained from the biological sample (Table 2). Test arthropods contained springtails (Collembola), mites (Acari), coleopterans (Coleoptera), dipteran larvae (Diptera), isopods (Isopoda), and diplopods (Diplopoda). For the semi natural tests, we did not use mesh above the collecting funnel of the device. Arthropods directly fell into it from the Berlese funnel, so other small arthropods could fly into the traps, above the collecting funnel. Arthropods directly fell into the device from the Berlese funnel. As a result, arthropods, such as Psocoptera and Curculionidae appeared in the sample as by-catch although in small numbers.

## 3. Results

### 3.1. Process Testing using the Desktop Model

The detection accuracy varied with the species (Table 1). The sensor ring detected 88% of *F. candida*, 94% of *H. nitidus*, 100% of *H. aculeifer,* and 100% of oribatid mite specimens.

After successful detection, microarthropods fell into the camera chamber. Seventy-eight percent of *F. candida*, 74% of *H. nitidus*, 92% of *H. aculeifer,* and 78% of oribatid mites were observable on the small-screen monitor attached to the camera. That is, these percentages of animals arrived adequately to the camera chamber (they did not get stuck or did not move out earlier) (Table 1).

**Figure 4:**
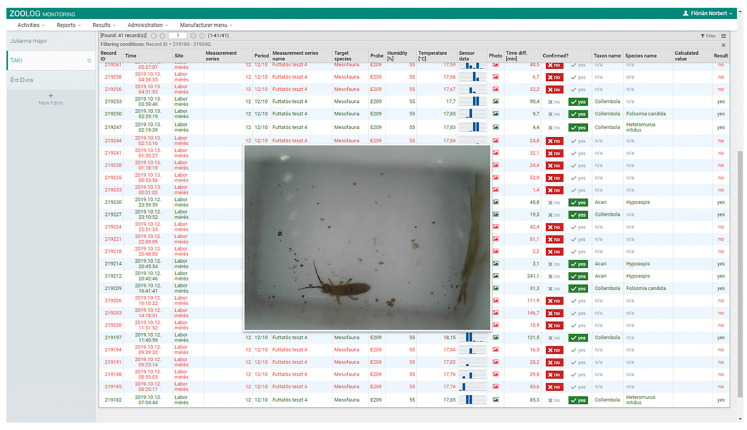
The user interface of the ZooLog Monitoring System. Each row corresponds to one detection. A photo is taken at each detection, which can be enlarged.

From those specimens that were previously successfully detected, 75% of *F. candida*, 74% of *H. nitidus*, 46% of *H. aculeifer,* and 50% of oribatid mites were successfully photographed and successfully identified from the data (and from jpg files) sent to the ZooLog Online web interface (https://helion.hu/zoolog/, Figure 4 and Table 1). These pictures were different in capture quality, depending on the body orientation and size of the microarthropods. However, for experts with a known biological sample, identification from these data is an easy and rapid process. Supplement 2 contains several pictures of 13 arthropod (mainly microarthropod) species. We present photos of good quality, from which specimens could be clearly identified, and also pictures of poor quality when arthropods could be identified by prior knowledge of the biological sample. 

From the camera chamber, previously detected arthropods were pumped out successfully at the first attempt in 61% of *F. candida*, 66% of *H. nitidus*, 88% of *H. aculeifer,* and 78% of oribatid mites (Table 1). These numbers are independent of the success of arriving into the camera chamber and also from photo shooting. The vacuum could pump out the specimens from the funnel, where they were stuck in or from other spaces, where the camera did not see them. These specimens could not be seen on the small-screen monitor or the pictures, but they came out into the container.

The failures of the specific processes (detection, entering the camera chamber, photo shooting, vacuum) could be added up. The whole process was correct in 42% of *F. candida*, 46% of *H. nitidus*, 44% of *H. aculeifer,* and 48% of oribatid mites (Table 1).

### 3.2. Semi-Natural Experiment

In the soil extraction experiment, where we applied soils with a known number of microarthropods, we gained back 77.0% of the specimens from the 656 specimens (Table 2). This ratio varied with species. The rate of captured microarthropods counted in the sample container (505 specimens) to which a picture could be assigned (330 specimens) was 65.3%, on average, and also depended on species morphology and size (Table 2).

## 4. Discussion

The continuous automatic monitoring of insects, and especially monitoring their activity, was previously investigated in many research papers [24]. Concerning soil- or surface-living insect species, two types of methods have been applied: opto-electronic sensors and digital cameras. Besides these “classical” techniques, passive acoustics was also used in monitoring studies [25]. While opto-electronic sensors have low power consumption and are less species-specific, cameras are just the opposite: They have a high energy demand and improved image analysis techniques for species determination [26]. There are other unique methods, such as the time-sorting pitfall trap, which records insects only for a limited time frame (e.g., days or dayparts) [16]. Technological improvements in the last decade provided, for example, outdoor cameras which previously had a high energy consumption. Nowadays, new energy-saving devices are already affordable for ecological studies. For example, time-lapse cameras programmed with 1- or 15-min intervals operate with batteries, even for up to four months [19]. Opto-electronic sensors were used in our earlier probe development [20]. Sensor data were biased, especially in arid, sandy soil where sand particles could fall into the probe. Since fall-in events between insects and soil sand particles could not be distinguished, the frequency of detection was higher than the specimens captured [27]. The new camera-supported probe presented in this paper combines pitfall and camera-traps. Our idea was that the camera should be switched on only when an arthropod enters the trap by using an opto-electronic infrared sensor. Therefore, the energy consumption is decreased compared to earlier methods, while the monitoring can function continuously. The opto-electronic infrared sensor ring could detect microarthropods with 73%-100% accuracy, depending on morphology and body sizes [22].

Photographing of trapped insects provides an opportunity both to decrease the number of false detections (e.g., detected soil particles can easily be filtered out based on the pictures) and to classify the detected microarthropods into higher taxonomic groups. The picture quality is sufficient to accurately identify the main groups of microarthropods. After captured microarthropods are identified from the biological samples, some species can be linked to their pictures, as well. The level of the distinctness in the photos highly depends on the given taxonomic group.

At the current stage of device development, the accuracy of the whole process (from detection until photo-sending) was 35–52% (44% on average). Most of the accuracy problems originated from two occasions.

First, microarthropods are not photographed at all. The number of pictures made from microarthropods was lower than the number of microarthropods captured in the sampling container. The timing of photographing (3 seconds after detection) was optimized for the average moving time of microarthropods. However, animals sometimes arrived in the chamber just following photographing, especially if they were stuck in the funnel. At that time, the vacuum pumped them out from the camera chamber without taking a photo. Other failures were observed by especially motile specimens, which quickly left the camera chamber before they would have been photographed. They could also climb up to the blind zone, back to the funnel, or even to the vacuum pipe. 

Second, microarthropods are photographed with some delay. In the process tests, 19.5% of the microarthropods did not reach the camera chamber in time because they clung onto the wall of the outer (less often) and the inner funnel (most often); however, it depended on the arthropod species. For example, some Acari, especially smaller carnivorous species, can easily attach to different surfaces. These microarthropods will arrive in the camera chamber, but usually they will be photographed and pumped out at the next detection when another in-falling object activates the system. Therefore, data generated by these specimens will be recorded in the database with some delay.

The accuracy results of the process test and semi-natural experiment were different. In the semi-natural experiment, better results were obtained, comparing the numbers of arthropods in the biological sample to the photos, almost 66% of the specimens were photographed. In that case, we do not know if the specimens were photographed and pumped out immediately or if they remained in the camera chamber for a longer time. Since in-falling soil particles also turned on the whole process (detection, photo shooting, vacuum), microarthropods that were stuck could be easily photographed and pumped up to the sample container in the next detection. For the process test, we did not consider the delayed photographing as part of a successful process and it had lower accuracy results. Therefore, we could gain better ratios for the semi-natural experiment while comparing the photos taken of the microarthropods to the specimens found in the biological samples.

Long-term use of pitfall traps may deplete the local fauna [28]. Our trap can be switched off and closed for months. Thus, it allows long-term monitoring when the research focuses on short periods. In addition, the new probes can be placed into the soil more quickly, as the mechanical part is shallower than in the case of previous EDAPHOLOG probes. Since the sample container can be placed aboveground, after digging down the probe into the soil, it can run without further considerable environmental disturbance. 

Berlese funnel tests showed that our device has the potential to be used in field conditions. This camera-supported probe is suitable to be used at the surface and under pitfall traps with adequate protection against objects falling in and arthropods of larger size. The probe can also be sunk into the soil to measure the activity of soil-living arthropods, where a particular catching part is needed to be designed. This device provides continuous, time-series data about the activity patterns of soil microarthropods throughout seasons. In contrast to data obtained from classical pitfall traps, which produced weekly or monthly frequencies, the new probe provides very dense data in time, supplying new opportunities for studying and understanding insect dynamics.

## 5. Conclusions

By using this newly developed probe, there is an excellent opportunity to get more detailed data about epigeic and hemiedaphic microarthropod species. With the new process, combining the advantages of the opto-electronic IR sensor and camera trap, a more precise estimation of activity time can be achieved.

By taking pictures of microarthropods, the taxonomical shortage of automatic detection can be resolved, and some level of species specificity can be available.

The collecting funnel at the top of the probe functions as a pitfall trap: epiedaphic (ground-living) microarthropods walking on the ground fall into the funnel and are caught. In a later development, the probe can be lowered into the soil to trap soil-living microarthropods. In this case, a trapping part will be attached above the funnel.

## Figures and Tables

**Table 1:** Results of the accuracy of the desktop model. Results of the three species cultured in the laboratory are reported according to their body sizes; numbers of specimens are in brackets. Detection accuracy is defined as the mean of the number of detected specimens compared to the total specimens dropped into the probe. “Appears in the camera chamber” indicates the percent of the specimens entered into the camera chamber compared to the total number of specimens. In the next second two columns, percentages of the specimens vacuumed out and seen in the pictures are given compared to the number of detected specimens. The last column shows the percentage of the individuals which fulfilled the whole process.

Microarthropod Species	100% = Total Specimens		100% = Detected Specimens		The Whole Process Is Perfect
	Detection Accuracy	Appears in the Camera Chamber	Vacuum Function	Seen in the Sent Picture	
*F. candida* (50)	**88%**	**78%**	**75%**	**61%**	**42%**
<1 mm (22)	73%	82%	100%	63%	45%
>1 mm (28)	100%	75%	61%	61%	39%
*H. nitidus* (50)	**94%**	**74%**	**74%**	**66%**	**46%**
<1 mm (17)	82%	71%	71%	57%	35%
>1 mm (33)	100%	76%	76%	70%	52%
*H. aculeifer* (50) <1 mm	**100%**	**92%**	**46%**	**88%**	**44%**
Oribatida (50) <1 mm	**100%**	**78%**	**50%**	**78%**	**48%**

**Table 2:** Results of the semi-natural extraction test. Microarthropods were placed in defaunated soil and were extracted in Berlese funnels equipped with the probes. We recorded the number of specimens in the sample container of the probe and the number of specimens recognized in the photos. The ratio of these two values is shown in the last column (% of specimens in photos), which indicates the accuracy of the sensing.

Species/Taxonomic Groups	N. of Extraction	Sum of Specimens in Sample Container	Sum of Specimens in Pictures	% of Specimens in Photos (Mean ± SD)
Collembola	13	269	166	56.5 ± 28.9
*Heteromurus nitidus*	13	113	68	60.6 ± 21.5
*Folsomia candida*	13	95	60	66.2 ± 20.7
*Entomobrya*	8	13	11	75.0 ± 46.3
*Orchesella*	8	10	7	58.3 ± 49.6
*Tomocerus*	2	6	5	83.3 ± 23.6
*Lepidocyrtus*	4	6	5	68.8 ± 47.3
*Symphypleona*	2	2	0	0.0 ± 0.0
*Proisotoma*	3	24	10	39.6 ± 22.0
Acari	13	215	153	66.4 ± 27.1
Oribatid mites	13	101	73	77.6 ± 21.6
*Hypoaspis aculeifer*	13	100	70	71.4 ± 16.5
Other Acari	4	5	2	25.0 ± 50.0
Trombidiidae	6	9	8	91.7 ± 20.4
Isopoda	10	12	7	43.3 ± 47.3
Diplopoda	4	3	1	12.5 ± 25.0
Diptera larvae	4	1	0	0.0 ± 0.0
Coleoptera	6	5	3	50.0 ± 57.7
other Coleoptera	3	2	1	33.3 ± 57.7
Curcolionidae	3	3	2	66.7 ± 57.7

## Supplementary Materials

The following are available online at https://www.mdpi.com/2075-4450/11/4/244/s1, Supplement 1: Electronic construction of the camera and the connection of the sensors Supplement 2: Pictures of the various taxa photographed by the new camera sensor.
