# Supplementary material for: Detecting Soil Microarthropods with a Camera-Supported Trap
Source: Insects. 2020 Apr 14;11(4):244. doi: 10.3390/insects11040244 (PMC7240604; doi:10.3390/insects11040244)
Supplement: Supplementary file 1 [file insects-11-00244-s001.zip › Supplement 1.docx]

**Appendix 1.**

**Electronic construction of the camera and the connection of the sensors**

**Figure 1.** Wiring diagram of the camera shooting system.

**Figure 2.** Wiring diagram of the camera unit to GSM/GPRS communication

**Integrating the *4D SYSTEMS µCAM-II*** **type camera to the system**

The central unit controls the camera via the synchronous serial line. The CPU has four synchronous line communication units, but they only can be programmed by reconfigurable units. The communication of the camera has to be configured to CPU programming input. The camera uses the UART3 serial line.

The speed of the serial port, beside the 7,3728 MHz clock signal of the CPU is the highest standard maximum possible value: 921600 bit/sec. In case of dealing with a big amount of data, this speed can only be handled by DMA. To control the UART3 serial port, we use the DMA1 channel of the CPU.

The 4D SYSTEMS µCAM-II built into the system can convert the exposed pictures into JPEG files, and it is also able to store them in its own data storage till the CPU asks for it by using slow communication.

For minimizing electricity consumption, the camera automatically sets itself to standby after 15 seconds of inactivity. To take a picture, we can restart the camera with a command that takes about 3 seconds. The “SYNC” command that starts the camera again consists of 0xAA 0x0D plus 4 0x00 byte series (each command is 6 byte). The starting 0xAA byte before each command ensures the baud-rate detection for the electricity of the camera.

| 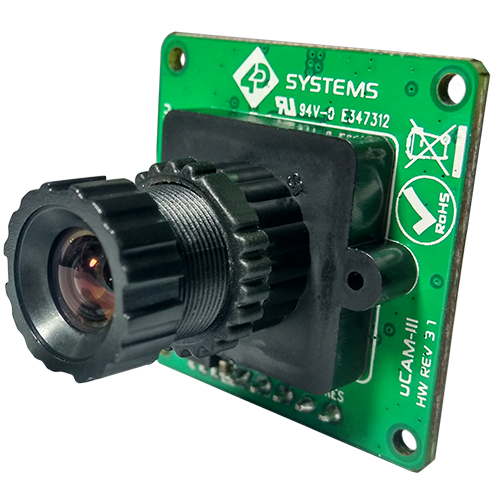 | 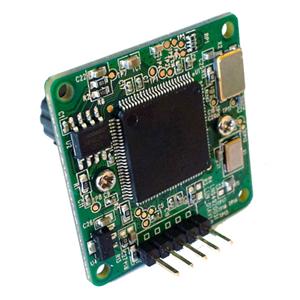 |
| --- | --- |

**Figure 3.** The 4D SYSTEMS µCAM-II type camera

After the “SYNC” command, the camera responds with an “ACK” byte series (0xAA 0x0E 0x0D) and a “SYNC” sequence. The CPU acks it with an “ACK” response. From this moment, the connection between the camera and the CPU is complete, and the camera can receive complete commands. First, the CPU sends an “INITIAL” command that sets the resolution, picture format, and the color depth of the recording. The basic value is 640x480, JPEG picture.

In the next command, we can set the size of the data blocks; the basic setting is 64 byte. The program uses this value because this fits the most for the abilities of the used microcontroller. Directly after ack this command, we can start the picture recording with the “SNAPSHOT” command (0xAA 0x05). We need another command for demanding the picture data. This is the “GET PICTURE” command (0xAA 0x04), for which the answer will arrive at about 70 ms after the demanding. After another 70 ms, it will send the information containing the length of the picture in the ”DATA” command (0xAA 0x0A). The CPU sends the ack “ACK” and prepares for receiving (via DMA) and storing the data from the camera.

The data traffic is controlled by the CPU. During the traffic, it writes out the received data every 256 bytes to the EEPROM, also using DMA via the SPI serial line. The 256-byte sorting is inevitable for the EEPROM because it can only receive this amount of data at the same time. It is subservient to use this amount because the CPU is not able for the much higher puffer.

**Figure 4.** The format of the data packages sent by the camera

******

**Figure 5.** The CPU unit of the camera probe

**Figure 6.** The parameters of the videocamera the 4D SYSTEMS µCAM-II type camera
