# Supplementary material for: Detecting Soil Microarthropods with a Camera-Supported Trap
Source: Insects. 2020 Apr 14;11(4):244. doi: 10.3390/insects11040244 (PMC7240604; doi:10.3390/insects11040244)
Supplement: Supplementary file 1 [file insects-11-00244-s001.zip › Supplement 2.docx]

**Appendix 2**

**Pictures of the various taxa photographed by the new camera sensor**

| **Taxa** | **Good quality pictures** | **Bad quality pictures** |
| --- | --- | --- |

| *Heteromurus nitidus,* Templeton (Collembola, Entomobryidae) | 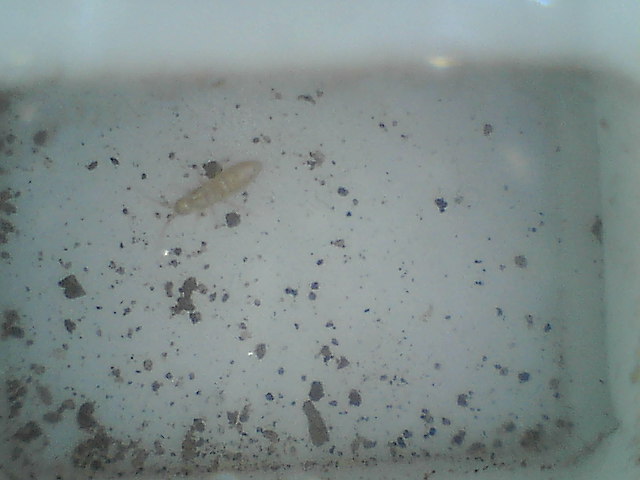 | 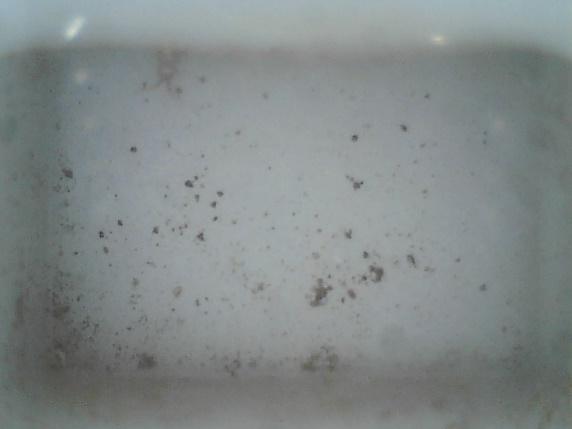 |
| --- | --- | --- |
| *Folsomia candida,*  Willem (Collembola, Isotomidae) | 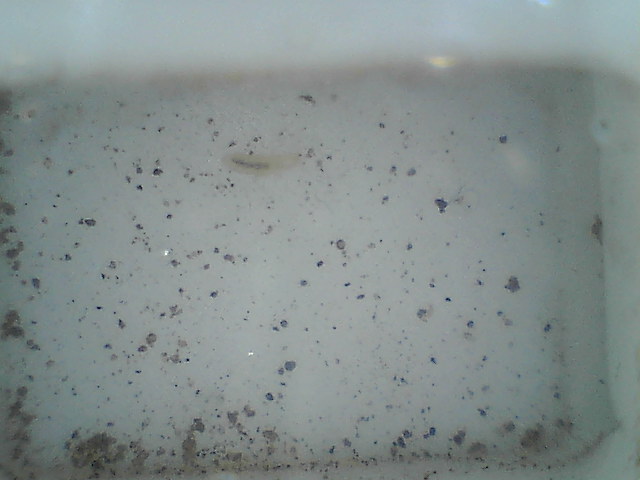 | 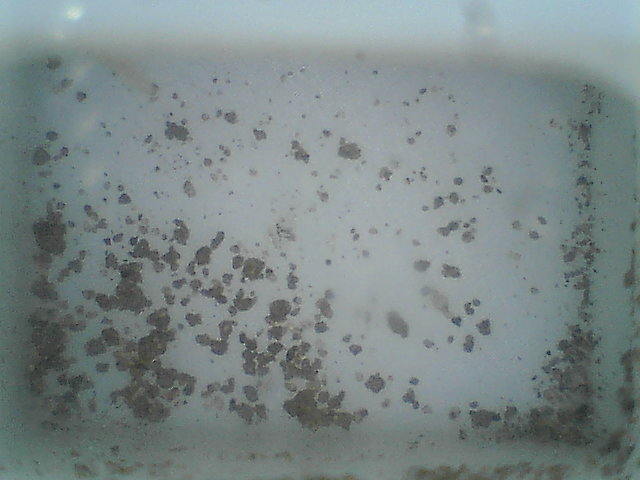 |
| *Orchesella cincta,*  Linnaeus  (Collembola, Entomobryidae) | 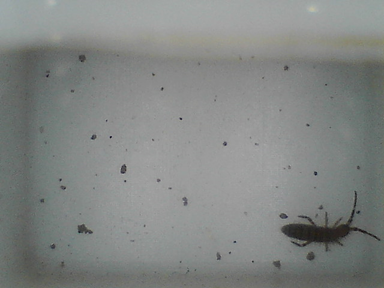 | 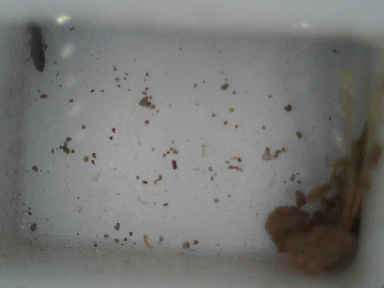 |
| *Entomobrya sp.,*  Rondani (Collembola, Entomobryidae) | 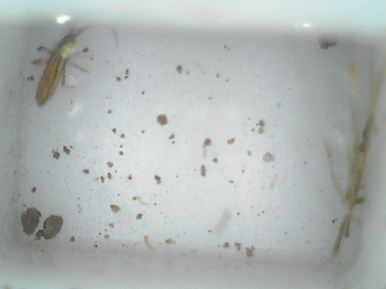 | 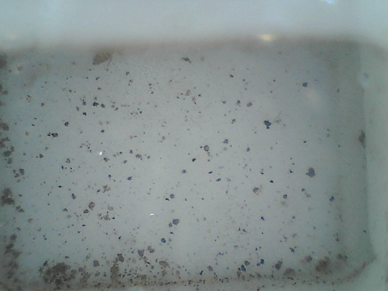 |
| *Lepidocyrtus sp.,*  Bourlet  (Collembola, Entomobryidae) | 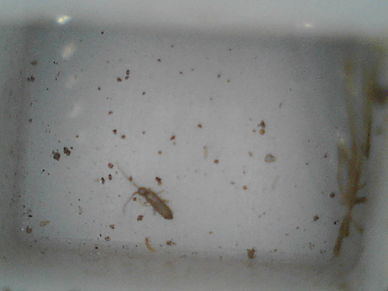 | 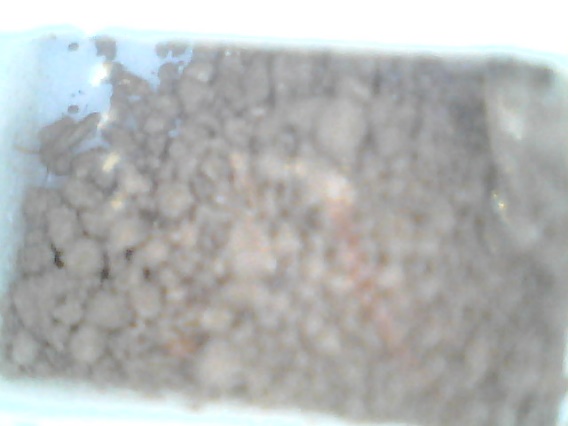 |
| *Proisotoma sp.,*  Börner  (Collembola, Isotomidae) | 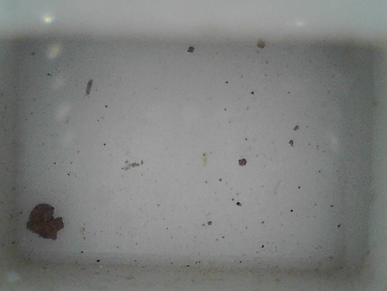 | 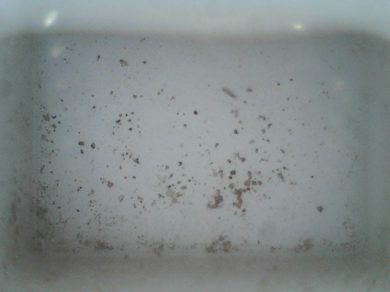 |
| *Tomocerus sp.,* [Nicolet](https://en.wikipedia.org/wiki/Hercule_Nicolet)  (Collembola, Tomoceridae) | 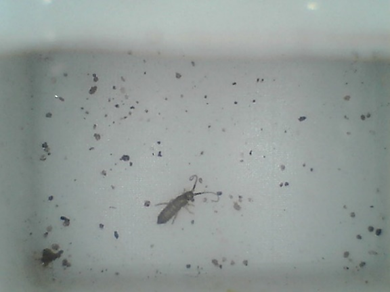 | 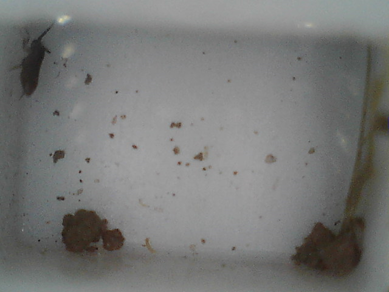 |
| *Trombidium sp.,*  Fabricius  (Acari, Trombidiidae) | 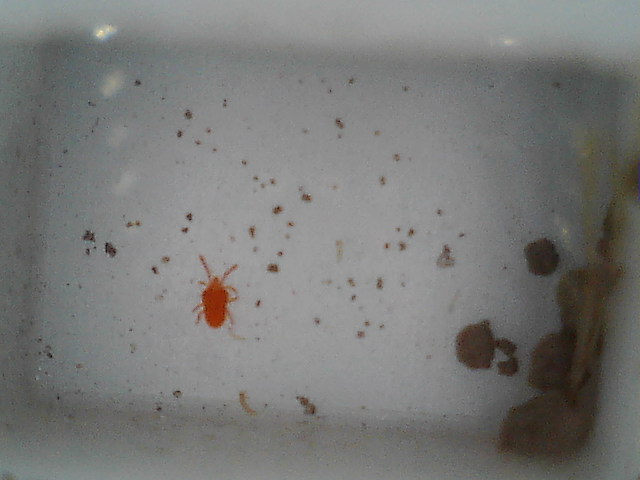 | 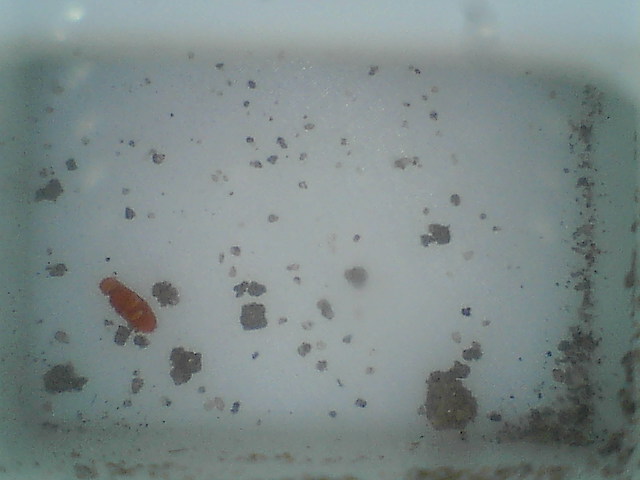 |
| *Hypoaspis aculeifer,* Canestrini (Acari, Laelapidae) | 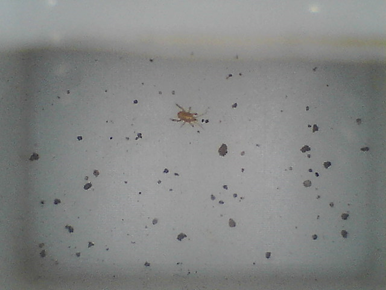 | 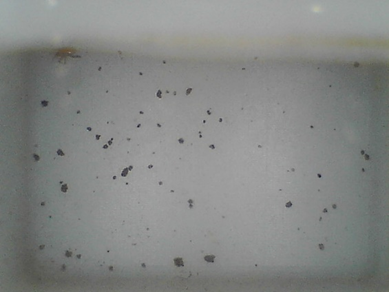 |
| *Oribatida sp.,*  Dugés  (Acari) | 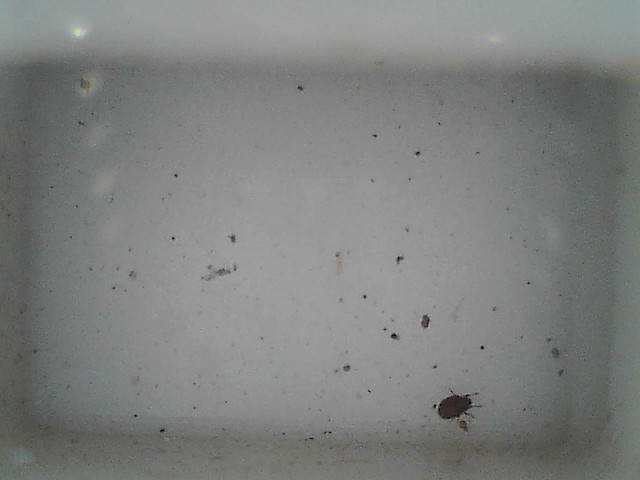 | 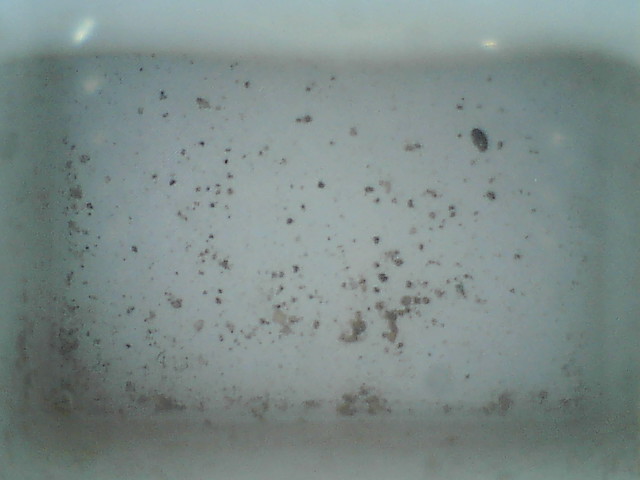 |
| *Isopoda sp.,*  Latreille  (Eumalacostraca) | 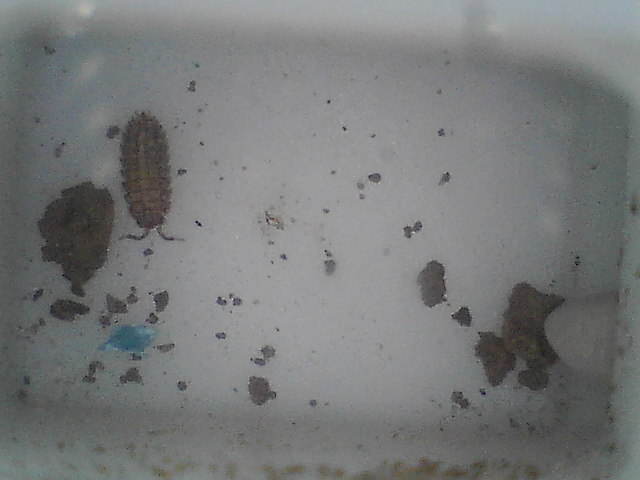 | 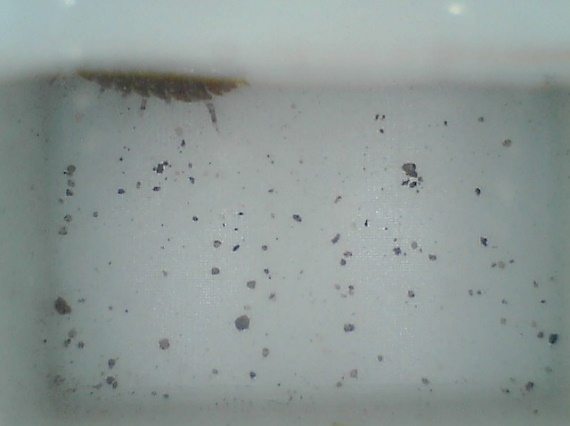 |
| *Psocoptera sp.,*  Haliday  (Pterygota) | 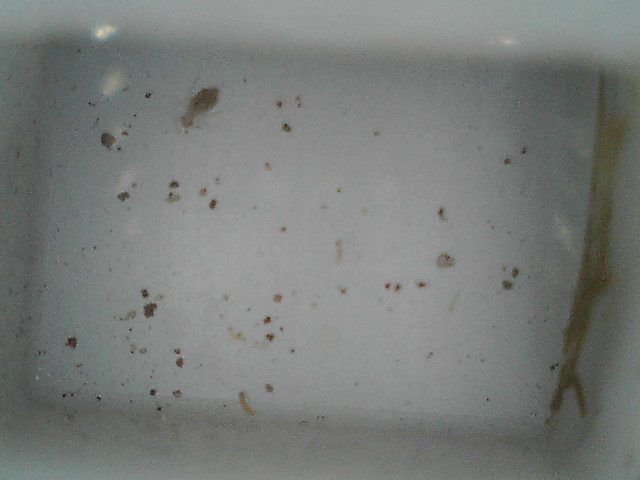 | 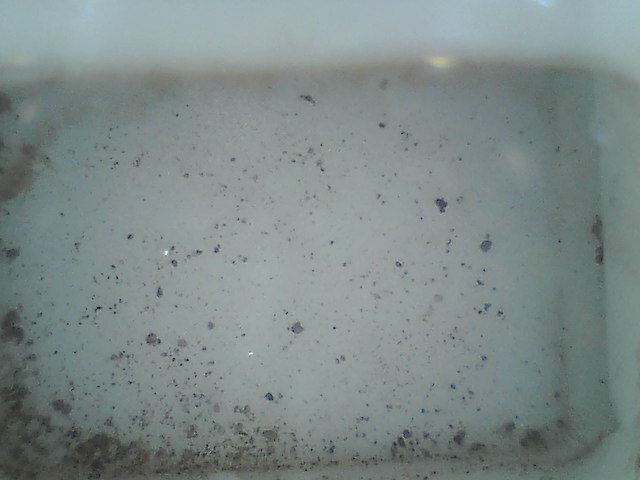 |
| *Curculionidae sp.,*  Latreille  (Coleoptera) | 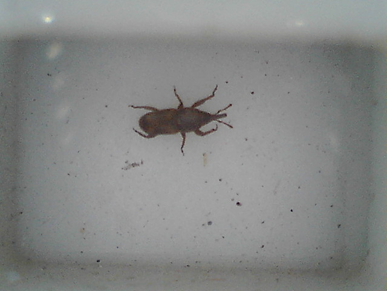 | 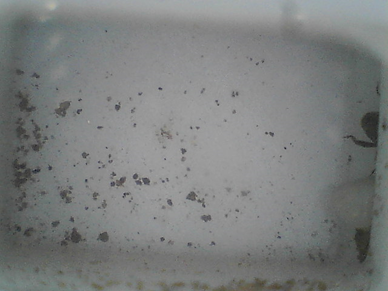 |
